# Effect of pre-supplementation with *Pleurotus sajor-caju* crude extracts on body weight and consequence responses of leukocytes and immune organs in fancy carp following inoculation with *Aeromonas veronii*

**DOI:** 10.14202/vetworld.2020.1010-1016

**Published:** 2020-05-31

**Authors:** Sitthichon Rattanachan, Sumrarn Bunnajirakul, Darsaniya Punyadarsaniya

**Affiliations:** 1 Clinic for Aquatic Animals, Faculty of Veterinary Medicine, Mahanakorn University of Technology, Bangkok, Thailand; 2 Department of Microbiology, Faculty of Veterinary Medicine, Mahanakorn University of Technology, Bangkok, Thailand

**Keywords:** *Aeromonas veronii*, body weight, fancy carp (*Cyprinus carpio*), immunostimulant, *Pleurotus sajor-caju*

## Abstract

**Aim:**

The present study aimed at highlighting the effects of oyster mushroom (*Pleurotus sajor-caju*), as a dietary supplement on growth performance, differential leukocytes population, and histological changes of melanomacrophage centers (MMCs) in spleen and kidney of fancy carp on bacterial infection.

**Materials and Methods:**

A total of 60 fancy carp were allocated into four groups according to feed formulations including; (1) basal diet with 2% crude extract of *P. sajor-caju*, (2) basal diet with 2% b-glucan, whereas Group 3, and Group 4 were positive and negative control, which were fed only basal diet. Diets were provided for 30 days, thereafter, fish of Group 1 to Group 3 were intraperitoneally injected with *Aeromonas veronii* (1.8×10^9^ CFU), whereas Group 4 was injected with normal saline. At day 7 post-bacterial inoculation, all fish were weighed, whole blood was collected for differential white blood cell count, and two visceral organs, posterior kidney and spleen, were collected from euthanized fish to observe histological changes, particularly MMCs.

**Results:**

No significant differences in body weight were found (p>0.05) at 1^st^ week of the experiment; however, fish body weight was significantly increased from week 2 to week 4 of the experiment. Increased monocyte number was found in carp fish fed with the *P. sajor-caju* or b-glucan supplemented diets compared to the control groups (p<0.05). The proliferation of monocyte in fish was consistent with increased number and size of MMCs in hemotopoietic organs, posterior kidney and spleen, especially in fancy carp fed with of *P. sajor-caju* crude extract and commercially available b-glucan before bacterial inoculation in fish.

**Conclusion:**

These findings indicate that crude polysaccharide from *P*. *sajor-caju* can be potentially used as a feed additive that might promote innate immune function in fish.

## Introduction

Immunostimulation in fish is induced by providing some substances to enhance the functional roles of innate immune response and to promote the endogenous defense systems. Therefore, the application of immunostimulants in fish farming might be very useful since it could offer protection against infection by aquatic pathogens with subsequent reduction of expenses related with the use of chemicals or medicines. As a result, sources of immunostimulating substances are nowadays extensively investigated. These kinds of materials can be produced synthetically or can be isolated from natural sources with different effectiveness. However, there are concerns about the possible toxic effects of synthetic drugs and chemicals on humans and animals [1]. Furthermore, excessive chemicals or drugs use for the control of infectious diseases in aquaculture farms can also cause negative effects on the aquatic environment [2]. In contrast, the application of natural immune promoting substances might be less toxic, less expensive, without inducing accumulation in animals and environment [3], thus use of natural immunostimulating dietary additives in fish farm can be beneficial for sustainable aquaculture.

Mushrooms is a kind of fungi that have been considered to be a source of food for human and animals since mushroom contains adequate levels of total carbohydrates, dietary fiber, protein, nitrogen, and ash [4]. In addition to their nutrients, mushrooms have been used as a traditional medicine due to their biological components, particularly immunomodulators such as terpenoids, lectins, fungal immunomodulatory proteins, and polysaccharides, which can trigger inflammatory processes and stimulate the activity of white blood cells that are involved in protecting organism against pathogens [5]. Some polysaccharides, such as beta glucan (b-glucan), possess beneficial properties including anti-inflammatory, antitumor, antimicrobial, antidiabetic, and immunostimulative activities [6].

To evaluate the effectiveness of mushroom as an alternative source of nutrient promoting of health benefits in fish; therefore, this experiment aimed to highlight the effects of edible oyster mushroom (*Pleurotus sajor-caju*), as a dietary supplement on growth performance, differential leukocyte profile, and histological changes of some visceral organs in fancy carp (*Cyprinus carpio*).

## Materials and Methods

### Ethical approval

This study was approved by the Ethics Committee of the Animal Care and use Committee of Mahanakorn University of Technology (Approval no. ACUC-MUT-2018/002).

### Mushroom preparation and extraction

Freshly purchased oyster mushrooms, *P. sajor-caju*, were cleaned by rinsing with tap water and minced into small pieces. Then, all minced mushrooms were air-dried in a hot air oven at a temperature at 40°C for 3 days to ensure a complete drying [6]. To obtain a crude extract of oyster mushroom, a hot water extraction method [7,8] partially modified was performed. Dried minced mushrooms were ground into a fine powder and subsequently combined with distilled water at a ratio of 1:20 and later boiled in a water bath at the temperature at 70°C for 5 h. The mixture was then filtrated through a sterile nylon sieve 2 times to remove dirt and the supernatant was collected and sent to central instrument facilities, Faculty of Science, Mahidol University. It was then lyophilized to obtain freeze-dried crude extract of oyster mushroom. This lyophilized crude extract was then stored at −20°C until further use.

### Measurement of total carbohydrate and total protein

Total carbohydrate levels of crude oyster mushroom extract were measured using the total Carbohydrate Quantification Assay Kit (Abcam, United Kingdom), according to the kit instructions. Total protein content of crude extract was also determined by Bradford assay [9,10] using Bradford reagent (AppliChem, Darmstadt, Germany), according to manufacturer’s instructions with bovine serum albumin for a standard protein.

### Fish and experimental design

A total 60 healthy fancy carps (*C. carpio*) with average body weight of 35 g and total length of body is 10 cm, which were purchased from local fish aquarium, were housed in glass tanks with aeration supplied for 7 days of the acclimatization. During this period, all fish were fed by commercial standard diet by 3% of body weight/fish, 2-times a day. After a period of acclimatization, all fish were divided into four major groups regarding to each experimental condition as following, (1) basal diet with 2% crude mushroom extract, (2) basal diet with 2% b-glucan, whereas Group 3 and Group 4 were positive and negative control, which were fed only basal diet, respectively, each major group was replicated as three small groups comprised five fish per group (total n = 15 fish/condition). During 30 days of the experiment, water parameters include temperature, dissolved oxygen, chlorine, pH, ammonia, and alkalinity were weekly detected (data not shown).

### Preparation of supplemented diets and feeding regime

The crude mushroom extract supplemented diet was prepared by incorporating 2% crude extract coated by vegetable oil into the diet [11] and was air-dried before its provision to the fish. The b-glucan supplemented diet was also prepared as previously described [11]. All experimental groups were fed twice per day continuously for 30 days.

### Bacterial preparation and experimental inoculation

*Aeromonas veronii* biovar sobria, which was isolated from naturally infected fish in a farm, was identified by the Department of Medical Sciences, Ministry of Public Health, Thailand. Thereafter, bacteria were maintained in MacConkey agar (Oxoid™, United Kingdom). A single colony of bacteria on the agar was then grown in Luria Bertani (LB) broth with 1% NaCl and kept at 37°C for 18 h. The amount of bacterial was quantitated as colony-forming unit per mL (CFU/mL) by agar plate dilution method. A stock of suspended bacteria was subsequently diluted with LB broth until the amount of bacteria were at approximately 1.8×10^9^ CFU/mL [12] and later washed 3 times with 0.9% sterile normal saline solution and finally suspended in 0.3 mL of normal saline before inoculation.

At day 30 of the experiment, experimental inoculation of fish by bacteria was carried out; all fish were sedated using clove oil as an anesthetic agent that was dissolved in 95% ethanol with a ratio of 1:10. Anesthesia of all fish was performed by short immersion in an anesthetic tank. All fish of Group 1 to Group 3 were then inoculated intraperitoneally with 0.3 mL of suspended bacteria, whereas fish from Group 4 were inoculated only normal saline solution. After inoculation, all fish were observed for 7 days to record their behavior and clinical signs due to the infection.

### Sample collection

During the feeding period, fish from each group were weekly weighed to measure body weight (g). Seven days after the experimental inoculation with bacteria, the blood samples were also collected from all experimental fish through the caudal blood vessel, and fish were then euthanized through the immersion for 10 min in water containing buffered tricaine methanesulfonate (MS-222, Merck, Germany), with a pH between 7.0 and 7.5. The blood samples from fish were smeared on a glass slide and stained using Diff-Quick staining set and later evaluated microscopically by differential white blood cell count for the number of neutrophils, monocyte, and lymphocyte. Then, all euthanized fish were dissected abdominally to remove spleen and kidney and subsequently preserved into 10% buffered formalin. These organs were then sent to MUT Veterinary Diagnostic Center for the examination of histological changes using a light microscope. The paraffinized tissue was embedded, sectioned, and stained by hematoxylin and eosin before the analysis.

### Statistical analysis

Body weight of fish is presented as group average body weight (g)±standard deviation (SD). Differential white blood cell count, number of neutrophils, monocyte, and lymphocyte are presented as min, max, and average (x̄)±SD. The above variables were evaluated by one-way analysis of variance using IBM SPSS Statistics version 23 (IBM Corp, Armonk, New York), Subscription Trial activated December 18, 2018. The probability level below 0.05 was considered significantly different.

## Results and Discussion

### *P. sajor-caju* and its crude extract

Our result demonstrated that crude extract of *P. sajor-caju* (oyster mushroom) can be prepared using the hot water extraction method as previously described [7] with slight modifications. In this study, 100 mg of crude mushroom extract contained 14.64 mg of total carbohydrate and 0.084 mg of total protein, respectively. This fact indicates that is feasible to isolate nutrients from oyster mushroom crude extract by the hot water extraction method. In our experiment, we did not detect any kind of nutrients that is associated with immune enhancement in mammals, particularly b-glucan for instance, however, as indicated by the previous experiment [7], the extraction of ground *Pleurotus pulmonarius* by this method can provide a high yield of crude protein (23.14 g per 100 g of mushroom dried weight) and crude carbohydrate (48.24 g/100 g of mushroom dried weight) and also b-glucan (20.05 g). The yield of crude carbohydrate and crude protein from oyster mushroom, in our study, was quite low compared to other studies using water as a solvent and various boiling temperature with additional enzyme for extraction [7,8,13]; however, a yield of crude polysaccharide obtained from mushroom depends on sample nature, extraction temperature, cooking pressure, pH, ionic strength of the solvent, and fineness of ground dried mushroom particle [14]. The analysis of crude mushroom extract is illustrated in Table-1 [7,11].

**Table-1:** *P. sajor-caju*, proximate parameters, and extraction condition.

Mushroom	*P. sajor-caju*^1^	*P. sajor-caju*^2^	*P. pulmonarius*^3^
Wet weight (g)	150	2000	ND
Dry weight (g)	31	222	100
Boiling water temperature (°C)	70	100	ND
Boiling time (h)	5	ND	5
Total protein	0.084 mg/100 mg of CE	ND	23.14 mg
Total carbohydrate	14.64 mg/100 mg of CE	11.4 g	48.24 g

1 Proximate data collected in this study,

2 Data obtained from Dobšíková *et al.* [11] and

3 Data obtained from Ahmed *et al.* [7]. CE=Crude extract, ND=Not described, *P. sajor-caju*=*Pleurotus sajor-caju*, *P. pulmonarius*=*Pleurotus pulmonarius*

### Effect of *P. sajor-caju* crude extracts dietary supplementation on fish body weight

In our experiment, fish mean body weight from each experimental group was weekly recorded, as shown in Table-2. At week 1 of the experiment, no significant differences in body weight among the four groups were observed. The mean body weight for negative controls, positive controls, fish supplemented with *P. sajor-caju* mushroom crude extract, and fish supplemented with b-glucan was 35.29±0.76 g, 35.29±0.76 g, 35.29±0.72 g, and 35.57±1.43 g, respectively. At week 2, the highest mean body weight was observed in fish fed the b-glucan supplemented diet (39.00±0.00 g) (p<0.05), whereas no significant differences in the body weight among three other groups were found (37.00±0.00 vs. 37.29±0.71 vs. 37.29±0.72 g for negative control, and positive control) and fish fed the mushroom crude extract supplemented diet, respectively (p>0.05). At week 3, the highest to lowest mean body weight was: Positive controls (39.71±1.89 g), fish fed the mushroom crude extract supplemented diet (39.50±1.25 g), fish fed the b-glucan supplemented diet (39.00±0.00 g), and negative controls (37.00±0.00 g). The same order was also observed in week 4 of experiment including; 44.00±0.01 g, 42.50±0.01 g, 39.00±0.01 g, and 37.00±0.06 g, respectively (p<0.05; Table-2). Interestingly, there was a slight effect of b-glucan on the body weight of fish, since the highest fish body weight was found in positive controls, which was fed only the basal diet. This result was consistent with other researches [15-17] and might be attributed to the lack of the digestive enzyme called b-glucanase, which is responsible for the digestion of b-glucan [18]. As compared to fish fed the *P. sajor-caju* crude extract supplemented diets, mean body weight of fish was higher to fish fed by supplemented feed with b-glucan but slightly lower than fish of positive control group, this might imply that fish can utilize heated water-soluble nutrients of *P. sajor-caju* since the crude extract we used in this experiment contains both crude polysaccharide (14.64 mg/100 mg of crude extract) and crude protein (0.084 mg/100 mg of crude extract) crude extract). Several factors can affect fish body gain such as source and type of carbohydrate, type of digestive enzyme, and fish species especially herbivorous or carnivorous [19]. In our experiment, crude extract of *P. sajor-caju* was used without isolation of b-glucan and removal of starch carbohydrate and protein, this implies that cyprinid fish can utilize low molecular carbohydrate components and also other nutrients from water-soluble crude mushroom extract by amylase enzyme [19]. In addition to digestion of crude extract derived from mushroom in fish, indigenous components of mushroom polysaccharides such as chitin, hemicelluloses, a- and b-glucan, mannans, xylans, and galactose are potential source of prebiotics [20], which can utilize by gut microflora that indirectly provide the host for energy, metabolic substrates, and essential micronutrients [21].

**Table-2:** Mean body weight (g) of carps per week.

Groups	Negative control	Positive control	Treatment 1 (mushroom)	Treatment 2 (β-glucan)	Significance*
Week 1	35.29±0.76 g^a^	35.29±0.76 g^a^	35.29±0.72 g^a^	35.57±1.43 g^a^	0.933
Week 2	37.00±0.00 g^X^	37.29±0.71 g^X^	37.29±0.72 g^X^	39.00±0.00 g^Y^	0.000
Week 3	37.00±0.00 g^A^	39.71±1.89 g^B^	39.50±1.25 g^B^	39.00±0.00 g^B^	0.001
Week 4	37.00±0.06 g^I^	44.00±0.01 g^II^	42.50±0.01 g^III^	39.00±0.01 g^IV^	0.000

* One-way ANOVA/different letters indicate significant differences (p<0.05) (Tukey’s HSD) at the same time period

### Effect of *P. sajor-caju* crude extracts dietary supplementation on fish differential leukocytes profile after the experimental infection with *Aeromonas veronii*

Health benefits effect of crude *P. sajor-caju* supplement in fancy carps on experimental infection with *A. veronii* bv. sobria at the 7^th^ day after inoculation was determined by manual differential leukocytes count through blood smear. In our study, no fish mortality was observed during experimental infection and the result of differential count (Table-3) shown that the population of neutrophils in negative controls (31.70±4.28 cells, 31.92%) and positive controls (28.00±7.52 cells, 28.57%) was greater than that of the groups dietary supplemented either with mushroom (10.30±2.31 cells, 10.29%) or with b-glucan (7.58±1.49 cells, 7.54%) (p<0.05). This finding might be related to individual health status and biological responsiveness to environmental parameters [22]; however, increased average number of neutrophils was found especially in fish of negative control group, which was fed by only standard diet and not inoculated by bacteria, might be due to individually stress response to surrounding environment [23]. In addition to environmental stress, there are some reasons that the number of neutrophils population was fluctuated as observed on the 7^th^ day after inoculation. Havixbeck and Barreda [24] indicated that high number of neutrophils in blood circulation are from 6 h to 24 h for the acute response to bacterial infection and undergo apoptosis once pathogen is eliminated and later modulate the function of macrophage to remove of apoptotic cells [25]. Furthermore, Charlie-Silva *et al*. [26] showed that the number of circulating neutrophils was decreased in tilapia infected with *Aeromonas hydrophila* in association with increased granulocyte accumulation in the inflammatory site. However, a high number of circulating neutrophils is possible in case of delayed apoptosis of neutrophils or impaired clearance of neutrophils at infection site and contributes to the prolong secretion of pro-inflammatory mediators such as granulocyte macrophage colony-stimulating factor, interleukin-8, or bacterial components [27]. For the population of lymphocyte, higher number of cells was also found in fish of positive control group (56.87±7.26 cells, 58.03%) and negative control group (53.37±3.16 cells, 53.74%) compared to fish supplemented with mushroom (39.68±5.11 cells, 39.56%) and b-glucan (35.83±2.83 cells, 35.65%) (p<0.05), this evidence is possible because of partial death of fish was found during housing before starting inoculation with bacteria. Nevertheless, number of lymphocyte observed among four groups of experiment seem to be usual since most studies have indicated that lymphocyte in common carp is the major population of white blood cell, which commonly found approximately 80% of leukocyte population [28]. For the number of monocytes, it was found to be higher in carp fish group both fed by *P. sajor-caju* (50.32±5.51 cells, 50.17%) and fish group fed by b-glucan (57.04±2.72 cells, 56.76%) compared to those in negative control group (14.23±3.67 cells, 14.33%) and positive control group (13.13±4.84 cells, 13.40%) (p<0.05); however, it is interesting that no significant difference in the number of monocyte was noticed between fish supplemented with crude *P. sajor-caju* extract and by b-glucan (p>0.05). This implies that 2% crude extract of *P. sajor-caju* can improve some of innate immune response in carp fish, and this finding was similar with the previous studies; for instance, Safari and Sarkheil [29] found that 1.5-2% dietary administration of *Pleurotus eryngii* induced higher number of white blood cells, particularly, monocyte, and activities of immunoglobulin, lysozyme, and complement at 63 days of the experiment. In addition, Minato *et al*. [30] demonstrated that polysaccharide of eatable mushroom, *Pleurotus citrinopileatus*, can modulate the differentiation of monocyte to macrophage. Interestingly, Minato *et al*. [30] proved that the competitive inhibition of dectin-1 and toll-like receptor-2 (TLR-2) receptors for b-glucan and polysaccharide on monocyte can affect the differentiation from monocyte to macrophage. In addition, Garcia-Valtanen *et al*. [31] showed that the supplementation of b-glucan during *in vitro* culture of monocytes from murine and human correlated to the increased viability and lifespan of these cells and influenced the differentiation from monocytes to macrophages at day 5 of the experiment compared to monocytes without b-glucan. These evidences suggest that crude polysaccharide from *Pleurotus* spp. has beneficial effects on immune system in humans and animals.

**Table-3:** Number of differential white blood cell count.

Observed white blood cell	Group	Number of animals per group	Number of differential white blood cell				Significance*

			Min.	Max.	Mean±SD	%	
Neutrophils	1	15	20	43	31.70±4.28^A^	31.92	0.000
	2	15	7	47	28.00±7.52^A^	28.57	
	3	14	6	19	10.30±2.31^B^	10.29	
	4	12	3	11	7.58±1.49^B^	7.54	
Lymphocyte	1	15	41	63	53.37±3.16^C^	53.74	0.000
	2	15	29	76	56.87±7.26^C^	58.03	
	3	14	21	55	39.68±5.11^D^	39.56	
	4	12	21	42	35.83±2.83^D^	35.65	
Monocyte	1	15	3	35	14.23±3.67^E^	14.33	0.000
	2	15	21	55	13.13±4.84^E^	13.40	
	3	14	50	70	50.32±5.51^F^	50.17	
	4	12	50	74	57.04±2.72^G^	56.76	

Group 1 (negative control), 2 (positive control), 3 (supplemented by *Pleurotus sajor-caju*), and 4 (supplemented by β-glucan)/

* one-way ANOVA/different letters indicate significant differences (p<0.05) (Tukey’s HSD) at the same time period

### Histological changes of kidney and spleen in fancy carp after the experimental infection with the *A. veronii*

**Figure-1:**
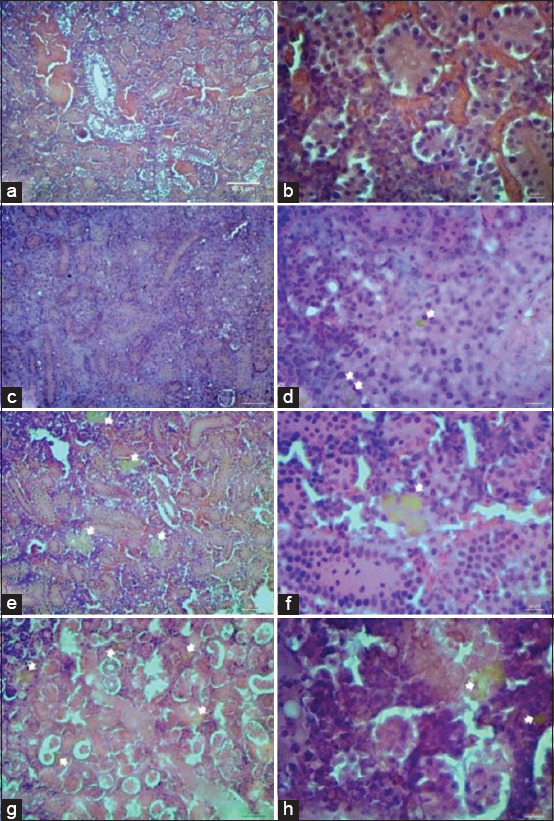
Histology of the posterior kidney (a, c, e, g, 100× power micrograph, b, d, f, h, 400× power micrograph) in fancy carp fish with four different feed supplements; negative control (a and b), positive control (c and d), fed with crude *Pleurotus sajor-caju* (e and f), fed with b-glucan (g and h). White arrows indicate melanomacrophage centers. Hematoxylin and Eosin staining.

**Figure-2:**
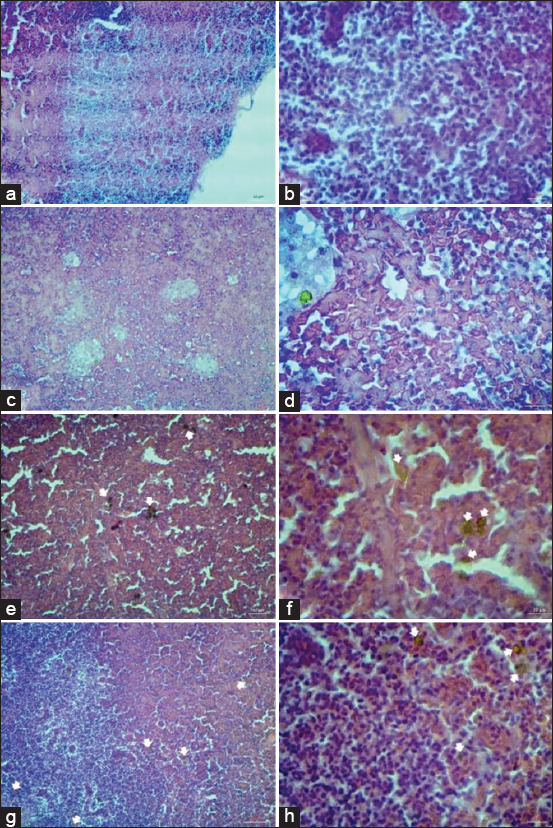
Histology of the spleen (a, c, e, g, 100× power micrograph, b, d, f, h, 400× power micrograph) in fancy carp fish with four different feed supplements; negative control (a and b), positive control (c and d), fed with crude *Pleurotus sajor-caju* (e and f), fed with b-glucan (g and h). White arrows indicate melanomacrophage centers. Hematoxylin and Eosin staining.

Kidney and spleen tissue sections were collected from fancy carp to compare the histological changes among the four experimental groups. The result revealed proliferation of melanomacrophage centers (MMCs, Figures-1 and 2), which are focal accumulations of macrophages that contain pigments, such as hemosiderin, lipofuscin, ceroid, and melanin in hematopoietic tissue of organs such as posterior kidney and spleen [32]. Alteration in amount and size of MMCs was obviously observed in fish fed the mushroom crude extract supplemented diet (Figure-1e and 1f, Figure-2e and 2f) and this finding was similar to that of fish fed with b-glucan supplemented diets (Figure-1g and 1h, Figure-2g and 2h), whereas a lower number of smaller MMCs was found in fish of positive control group (Figure-1c and 1d, Figure-2c and 2d), which was not fed by any supplement. However, no proliferation of MMCs was found in fish of negative control group (Figure-1a and 1b, Figure-2a and 2b). This finding indicates that feed supplement using crude polysaccharide from *P. sajor-caju* and b-glucan might activate MMCs. This is quite similar to the results of a previous experiment [33] that accessed the effectiveness of microalgae *Navicula* sp. orally administration on immune response in Pacific red snapper. Melanomacrophage was predominantly observed in the submucosa of intestine with the higher serum immunoglobulin M and this evidence could be possibly attributed not only to the high content of b-glucan and cell wall polysaccharide [34] but also to the activation of MMCs was noticed in fish injected with infectious agent or non-infectious agent to trap and scavenge foreign bodies [35]. In addition, transmembrane protein receptor called dectin-1, which found in monocyte and macrophage, was a specific receptor for b-1,3-glucan, and might associate with TLR-2 on macrophage for its activation in the presence of invading bacteria [36].

## Conclusion

Overall findings demonstrate that water soluble extract of *P. sajor-caju* can be prepared by boiling in water, and its main ingredient is the crude polysaccharide. Supplement of *P. sajor-caju* crude extract in feed might be associated with improved weight gain in fish and increase of blood leukocytes, particularly monocyte, after the experimental infection with *A. veronii*. Proliferation of monocyte in fish was consistent with increasing number and size of MMCs in hemotopoietic organs, posterior kidney and spleen, especially in fancy carp fed with of *P. sajor-caju* crude extract and commercially available b-glucan before bacterial inoculation in fish. These evidences imply that crude polysaccharide from *P. sajor-caju* contribute to upregulation of innate immune function in animal. However, further investigations such as isolation and characterization of active ingredient that can be beneficial to immune system in animal, for example, glucans, for instance. In addition, initiation of phagocytic activity for monocyte and macrophage induced by the addition of crude polysaccharide isolated from *P. sajor-caju* should be further elucidated. Furthermore, cellular interactions of crude polysaccharide from mushroom and receptors on phagocytes and patterns of cytokines regulations of innate cells in response to invading pathogens are needed to clarify.

## Authors’ Contributions

SR was responsible for creating the research concept, conducting research, data collection and analysis, and drafting of the article. SB advised the experimental design and was also responsible for histological analysis of spleen, liver, and posterior kidney tissue samples. DP advised the experimental design and checking all figures of the article. All authors read and approved the final manuscript.
